# Discontinuation of Intermittent Hemodialysis in HIV-Associated Nephropathy Following Initiation of Antiretroviral Therapy

**DOI:** 10.7759/cureus.13181

**Published:** 2021-02-06

**Authors:** Mohamedanwar Ghandour, Hammam Shereef, Omeralfaroug Adam, Yahya Osman-Malik, Zeenat Bhat

**Affiliations:** 1 Internal Medicine/Nephrology, Detroit Medical Center, Wayne State University, Detroit, USA; 2 Internal Medicine, Beaumont Health, Dearborn, USA; 3 Internal Medicine, Detroit Medical Center, Wayne State University, Detroit, USA; 4 Nephrology, Detroit Medical Center, Wayne State University, Detroit, USA

**Keywords:** hiv-associated nephropathy, acquired immune deficiency syndrome (aids), antiretroviral therapy (art)

## Abstract

Human immunodeficiency virus (HIV) infection occurs due to the HIV virus. It results in an immunodeficient state and multi-organ system infections and malignancy known as AIDS. HIV-associated nephropathy (HIVAN) is the most common HIV kidney involvement and may present as acute kidney injury (AKI), as well as chronic kidney disease (CKD). HIVAN is a collapsing form of focal segmental glomerulosclerosis (FSGS). HIVAN treatment options include antiretroviral therapy (ART), steroids, angiotensin-converting enzyme inhibitors/angiotensin receptor blockers (ACEI/ARB), and hemodialysis (HD). We herein describe the case of a 40-year-old patient with an established diagnosis of HIVAN who has had partial recovery of end-stage renal failure following the initiation of ART.

## Introduction

Human immunodeficiency virus (HIV) infection is a multiorgan system disease that occurs due to decreased immunity. Kidney involvement is common and may present as acute kidney injury (AKI) as well as chronic kidney disease (CKD). However, HIV associated nephropathy (HIVAN) is the classic renal clinico-histological syndrome associated with uncontrolled HIV infection. It was first described in 1984 as a complication of acquired immune deficiency syndrome (AIDS) [1]. HIVAN is a collapsing form of focal segmental glomerulosclerosis (FSGS), which characterized histologically by microcystic tubular dilatation and interstitial inflammation [2]. Following the introduction of antiretroviral therapy (ART), the prognosis of kidney functions has shown significant improvement. Betjes and Verhagen reported improvement of renal function after the initiation of ART; however, the long-term renal function is not well-documented [3]. We present the case of a 40-year-old patient with an established diagnosis of HIVAN who has had partial recovery of end-stage renal failure following the initiation of ART.

## Case presentation

A 40-year-old African American female presented to ED for a sudden onset of left upper and lower extremity numbness which resolved on its own within one hour. Her past medical history included HIV diagnosed one month prior to admission for which she declined ART at that time. CT and MRI brain scans showed no acute process. Vital signs were within normal limits and the physical examination was unremarkable. Laboratory tests showed a plasma creatinine of 4.7 mg/dL, a blood urea nitrogen (BUN) of 41 mg/dL, and a glomerular filtration rate (GFR) of 12 mL/min/1.73 m^2^. A complete blood count (CBC) was remarkable for white blood cell (WBC) count of 2.8 k/cumm and platelets 146 k/cumm. No previous baseline creatinine was reported. Urinalysis revealed +3 protein. Renal ultrasound showed normal-sized kidneys. The right kidney measured 11.2 cm, and the left kidney measured 11.8 cm with normal echogenicity of the cortex.

Additional investigations revealed a positive HIV‐1 serology with a lowered cluster of differentiation 4 (CD4)‐positive lymphocyte count of 30/mm^3^ and a viral load of 250 copies/mL. Antinuclear antibody (ANA) and hepatitis B and C virus screening were negative with a normal C3 and C4. A renal biopsy performed and was consistent with HIVAN as per the patient. Her renal parameters continued to deteriorate, and the patient was started on intermittent hemodialysis through a permacath during the hospital course. A left upper extremity arteriovenous fistula (AVF) was created, and outpatient hemodialysis was arranged. Prior to discharge, the patient was started on ART (an antiviral regimen consisting of darunavir, ritonavir, tenofovir, and emtricitabine). A few months following ART initiation, the patient's renal functions started to improve and partially recovered; the creatinine went down to 2.6 mg/dL, and GFR 27 mL/min/1.73 m^2^. The patient was taken off dialysis and a decrease in proteinuria was noted as well (urine protein to creatinine ratio 1.4). The viral load dropped to < 48 copies/mL, and the CD4 cell count increased to 350/mm^3^.

## Discussion

HIV-associated nephropathy (HIVAN) is a disease of complex pathogenicity that manifests histologically as rapidly progressive collapsing focal and segmental glomerular sclerosis (FSGS) with subsequent tubular inflammation and interstitial edema and rapid development of renal failure correlating with the status of HIV disease [4-5]. HIVAN has a unique predilection to affect middle-aged people of Black descent, and it often necessitates the initiation of renal replacement therapy [6]. Many theories have been proposed to elucidate the pathogenesis in both clinical and pre-clinical models since HIVAN was first described in the mid-1980s.

A virus-related factor is thought to play a major role. Overexpression of HIV-1 genes, specifically Nef and Vpr in the kidney of mice, was associated with more progression of nephropathy [7-8]. On the other hand, a host of genetic vulnerability factors are believed to be contributing to the pathogenicity of HIVAN. Higher frequencies of myosin-9 (MYH-9) were initially thought to be linked to the host susceptibility to develop HIVAN. However, the apolipoprotein L1 (APOL1) gene is now believed to be the genetic factor [9-10].

Diagnosing HIVAN can be challenging as there is a broad differential for causes of acute and chronic kidney injury in HIV patients, including not only HIV-related causes but also renal toxicity caused by HIV antiretroviral agents. Consequently, a renal biopsy is often needed for the diagnosis [11]. HIVAN is associated with a broad range of glomerular and tubulointerstitial pathologies, the classic of which is the collapsing form of focal segmental glomerulosclerosis. Other features that can be seen include, but are not limited to, microcystic renal tubular dilatation, mesangial hypercellularity, podocyte hypertrophy, interstitial inflammation, fibrosis, and tubuloreticular inclusions [12].

The patient in this report showed the typical clinical and pathological characteristics of HIVAN. The patient is an African American with uncontrolled HIV, as evidenced by low CD4 lymphocyte counts and a high viral load. The patient showed a rapid decline in renal function with massive proteinuria.

The renal damage in HIVAN is usually rapid and can be irreversible. Recovery is thought to be dependent on the amount of glomerulosclerosis and interstitial fibrosis. The collapsed glomerulus with limited or no sclerosis may recover entirely [13]. In our case, no follow‐up biopsies were performed.

The main therapeutic options for the management of HIVAN are ART, proteinuria control with angiotensin-converting enzyme (ACE) inhibitors/angiotensin receptor blockers, and may involve steroid therapy for the suppression of inflammation [11, 14]. Given that the development of HIVAN can happen at any stage, treatment with ART remains the mainstay of therapy regardless of the CD4 count [14]. Several cases in the literature have been reported with evidence of improvement of renal function after the initiation of ART [3]. Even though there are no clinical trials explicitly addressing this issue, the Strategic Timing of Antiretroviral Treatment (START) trial has evaluated the association between kidney function measured by epidermal growth factor receptor (eGFR) and the initiation of ART at an immediate vs. later stage after disease diagnosis and found results showing higher eGFR levels and lower risk of proteinuria in the immediate treatment group [15]. Renal replacement therapy is not a treatment for HIVAN, but it certainly should be offered for patients progressing to end-stage renal disease (ESRD). In this subgroup of patients, renal transplantation can also be a therapeutic option [3].

The reported patient was initially started on intermittent hemodialysis for the rapid decline of her renal function. However, within 11 months of starting ART, the patient's GFR improved and kidney function recovered to the point that the patient no longer required renal replacement therapy.

## Conclusions

In summary, we have presented a case of HIVAN who was taken off dialysis following the initiation of ART. Further studies are encouraged to demonstrate the early correlation between ART initiation and liberation from dialysis dependence in a patient with HIV-associated nephropathy. We recommend early administration of ART in HIVAN patients, which may reverse progression to ESRD.
